# Associations between relative body fat and areal body surface roughness characteristics in 3D photonic body scans—a proof of feasibility

**DOI:** 10.1038/s41366-021-00758-w

**Published:** 2021-02-15

**Authors:** Severin Ritter, Kaspar Staub, Patrick Eppenberger

**Affiliations:** 1 Institute of Evolutionary Medicine, University of Zurich, Zurich, Switzerland; 2 Zurich Center for Integrative Human Physiology (ZIHP), University of Zurich, Zurich, Switzerland; 3 Center for Experimental and Clinical Imaging Technologies Zurich (EXCITE Zurich), Zurich, Switzerland

**Keywords:** Body mass index, Preclinical research

## Abstract

**Introduction:**

A reliable and accurate estimate of the percentage and distribution of adipose tissue in the human body is essential for evaluating the risk of developing chronic and noncommunicable diseases. A precise and differentiated method, which at the same time is fast, noninvasive, and straightforward to perform, would, therefore, be desirable. We sought a new approach to this research area by linking a person’s relative body fat with their body surface’s areal roughness characteristics.

**Materials and methods:**

For this feasibility study, we compared areal surface roughness characteristics, assessed from 3D photonic full-body scans of 76 Swiss young men, and compared the results with body impedance-based estimates of relative body fat. We developed an innovative method for characterizing the areal surface roughness distribution of a person’s entire body, in a similar approach as it is currently used in geoscience or material science applications. We then performed a statistical analysis using different linear and stepwise regression models.

**Results:**

In a stepwise regression analysis of areal surface roughness frequency tables, a combination of standard deviation, interquartile range, and mode showed the best association with relative body fat (*R*^2^ = 0.55, *p* < 0.0001). The best results were achieved by calculating the arithmetic mean height, capable of explaining up to three-quarters of the variance in relative body fat (*R*^2^ = 0.74, *p* < 0.001).

**Discussion and conclusion:**

This study shows that areal surface roughness characteristics assessed from 3D photonic whole-body scans associate well with relative body fat, therefore representing a viable new approach to improve current 3D scanner-based methods for determining body composition and obesity-associated health risks. Further investigations may validate our method with other data or provide a more detailed understanding of the relation between the body’s areal surface characteristics and adipose tissue distribution by including larger and more diverse populations or focusing on particular body segments.

## Introduction

The use of 3D photonic body scans (BS) to assess body shape in epidemiologic studies and for daily fitness tracking is on the rise, thanks to developments in equipment and measurement methods that provide fast, reproducible, and increasingly accurate results [1–7]. However, an essential limitation of many currently available approaches is the lack of a precise approximation of adipose tissue distribution, particularly in more obese individuals, despite its relevance for assessing the risk for metabolic and cardiovascular diseases [8–10]. Most established methods for assessing relative body fat (%BF) or body fat distribution in the clinical routine are suboptimal proxies for %BF or total body fat [11, 12]. Dual-energy X-ray absorptiometry (DXA) is the gold standard for assessing body composition and provides data on both volumetry and distribution of adipose tissue. Due to costs, duration, and ionizing radiation, it is not suited for the general clinical routine [13]. Several recent studies aimed to bridge this gap between volume, surface, and distribution of adipose tissue [14, 15], e.g., using multimodality registration of DXA data with 3D body surface scans [16], however, published data only included six individuals.

In order to reach a broad target population, measurement methods are needed, which provide a precise and differentiated assessment of %BF and, at the same time, remain straightforward for an application in daily clinical practice. Since this goal is still challenging to achieve, we sought a new approach to this research area by linking a person’s %BF with the roughness of the body surface. We recognize that the influence of the prognostically relevant visceral body fat on body surface characteristics may be somewhat limited. In contrast, the amount and distribution of subcutaneous adipose tissue, located between the musculoskeletal system and the skin, are likely to significantly influence body surface morphology and, thus, have a measurable impact on the areal surface roughness characteristics.

To test our approach as a possible starting point for a more accurate assessment of %BF, particularly subcutaneous adipose tissue, we evaluated the roughness of 76 test subjects’ body surfaces and compared the results with BIA estimates of their %BF.

## Material and methods

3D models used for our study were taken from a prior study in which the correlation of height and WC measured by BS and manual anthropometric measurements in young Swiss men was investigated [6, 17]. This prior study included a cross-sectional part and a re-examination 4 months later. The study was approved by the Cantonal Ethics Board of Zurich (No. 2016–01625), and all participants were required to sign a detailed form to ensure informed consent to later scientific evaluation of their data. Participation was voluntary, without further selection criteria such as origin, demographic factors, or socioeconomic status [6]. A semi-mobile 3D photonic full-body scanner (Anthroscan VITUSbodyscan, Human Solution, Kaiserslautern, Germany) was used for body surface data acquisition, providing spatial resolution of <1 mm, with a point density of 300 data points per cm^3^. Comparative %BF (relative body fat) estimates were assessed by BIA (Seca mBCA 515, Reinach, Switzerland). In the present study (Armed Forces basic training), it was not feasible to perform more extensive or time-consuming examinations than BIA. However, the technique and, more specifically, the Seca mBCA 515 device have been validated in various studies and have been widely used for benchmarking body composition measurements derived from photonic 3D BS [18, 19]. In a recent validation study, this device has shown high reliability in estimating total body fat [20]. WC was measured with a tension-stable hand-held tape measure with automatic retraction (Seca 201, Seca AG, Reinach, Switzerland). Height and weight were measured with a standard stadiometer (Seca 274, Seca AG, Reinach, Switzerland). Further details of the measuring protocol are given in prior studies [6, 17, 21]. Raw scan datasets—in a proprietary point cloud format—were later triangulated and closed using the standard scanner software (Anthroscan, Version 3.5.3, Human Solutions, Kaiserslautern, Germany) and exported to the .obj file format.

For our study, we used data from the cross-sectional part, for which 104 young male Swiss Armed Forces recruits were examined on the same day, using the same measuring protocol and equipment. We imported available 3D models of 104 study subjects into a 3D modeling software (Rhinoceros 3D 6.19, Robert McNeel & Associates, Seattle, WA, USA) to calculate total point count, volume, and surface area of each model. To analyze the surfaces of the 3D BS, in a similar approach as currently used for geoscience, material science, and further surface metrology-related applications [22], we used a multiplatform, open-source solution for point cloud analysis and editing (CloudCompare 2.9.1, Électricité de France SA, Paris, France). CloudCompare features a tool for areal surface roughness evaluation to analyze particular geometric characteristics of point clouds [23]. Unfortunately, 3D models generated for the original study had been exported and stored using two different resolution settings, and 28 out of 104 3D models had to be excluded from further analyses due to inconsistent resolution. The 76 included high-resolution 3D models contained a total of 425,691–594,259 points with a mean point count of 491,104 (±33,138). For each point of a point cloud, CloudCompare can calculate an areal roughness value according to the following steps:

1. A sphere with a user-defined radius is fitted around the point.
2. A best-fitting plane through all the points included in this sphere is determined.
3. The distance between the point (center of the sphere) and the best-fitting plane is calculated (areal roughness value).

From these values CloudCompare then creates an areal roughness histogram containing 256 classes, Class 1 being smooth (smallest distances) and Class 256 being rough (largest distances). We excluded Class 1 from statistical evaluations since it typically represented artificial flat surfaces of the 3D model, such as the foot sole. Also, since the absolute number of points contained in the point cloud models of our participants varied, absolute histogram values (number of points), calculated by CloudCompare, had to be divided by the total number of points contained within a particular point cloud, to obtain relative frequency tables and to be able to compare different 3D models statistically.

**Fig. 1:**
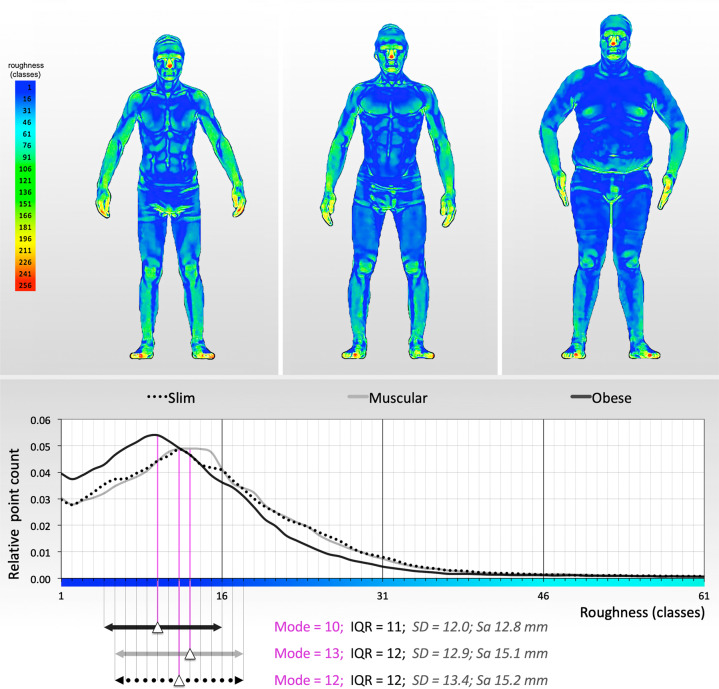
Roughness analyses mapped to three representative 3D surface models, with corresponding roughness frequency tables. Roughness analysis at radius = 2 cm (R2) mapped onto the 3D surface models of a slim (BMI = 19.5; relative body fat = 2.5%), a muscular (BMI = 21.0; relative body fat = 4.3%), and an obese (BMI = 30.1; relative body fat = 34.4%) study participant, illustrating the range of body shapes in our study sample, and corresponding roughness frequency tables with the mode and interquartile range (IQR) plotted as measures of central tendency and statistical dispersion, respectively. Standard deviation (SD) and arithmetic mean height (Sa), a quantitative roughness parameter (as defined by ISO 25178), are also given.

After a visual assessment (Fig. 1), we defined three different radii (R1 = 1 cm, R2 = 2 cm, R5 = 5 cm) and calculated three different areal surface roughness frequency tables for each of the 76 study subjects included in our study. Radii higher than 5 cm were not deemed appropriate since this way entire body parts were included in the calculation of best-fitting planes, rather than an area of the body’s surface. Also, some participants held their hands quite close to the trunk, resulting in the trunk and the upper extremities being mixed for the calculation of best-fitting planes when the radius exceeded 5 cm. Radii smaller than 1 cm revealed noise artifacts beyond the effective resolution of the scanner.

### Statistical methods

Descriptive statistics (mean, standard deviation (SD), minimum, maximum) of the basic parameters (age, height, weight, body mass index, etc.) were calculated for all included study subjects.

For further statistical analysis, the software R was used (R, 2.6.3, the R Foundation, https://www.r-project.org). In a first analysis step, we verified our initial assumption that the distribution of areal surface roughness levels across the 256 classes in the frequency tables is associated with %BF. For this, we computed different linear regression models (M1–M6) by the measures of statistical dispersion—SD and interquartile range (IQR)—for all three radius values (R1, R2, R5). After a visual assessment, we also assumed an association between the mode, defined as the class with the highest peak, and %BF. Therefore, further linear regression models, including the mode (M7–M9), were computed. Linear regressions have proved suitable for initial evaluations of associations between 3D photonic body scan measurements and body composition (with relatively small added value for nonlinear methods) [6, 17, 21].

In a second analysis step, to find the best association with %BF, we then combined SD, IQR, and mode in stepwise regression models for each radius (M10–M12). To investigate the importance of each explanatory variable (SD, IQR, mode) for %BF, we separately excluded each explanatory variable from the model and calculated the Akaike’s information criterion (AIC). Next, we calculated the difference between the AIC when excluding an explanatory variable and the AIC of the complete model. Therefore, the significance of the explanatory variable in the model corresponds to the differences of the respective AICs.

In a third and more explorative analysis step, to find an association between the overall roughness of each participant and %BF, we also calculated the arithmetic mean height (Sa), a quantitative roughness parameter (in mm), as defined by ISO 25178, frequently used in surface metrology. Sa is the extension of the concept of the arithmetic mean height of a line (Ra) to a surface. It expresses the average distance of any point of a surface to an arithmetic mean of that surface. For our application, we determined Sa by dividing the sum of all roughness values, as calculated for each point, by the total number of points in the 3D model. Therefore, Sa allows the comparison between participants, despite the point clouds containing different numbers of points, with normalization being part of the measure. Finally, linear regression models for the association between Sa and %BF (M13–M15) were also computed.

## Results

Descriptive statistics about the young men (*N* = 76) included in our study are displayed in Table 1. The participants were by average 20.5 years old (SD ± 1.1 years), 178.1 cm tall (±6.9 cm), and weighted 74.3 kg (±12.9 kg). Their mean BMI was 23.4 kg/m^2^ (±3.5 kg/m^2^), with 28% having excess weight (BMI ≥ 25 kg/m^2^), and 14% of those being obese (BMI ≥ 30 kg/m^2^) according to the WHO obesity classification (http://www.euro.who.int/en/health-topics/disease-prevention/nutrition/a-healthy-lifestyle/body-mass-index-bmi). According to BIA estimates, average relative fat mass (%BF) was 14.6% (±7.7%), and absolute fat mass was 11.7 kg (±8.0 kg). Linear regressions showed that BMI was able to explain a high share of the variability in %BF (*R*^2^ = 0.81, *p* < 0.001), the same applied for WC (*R*^2^ = 0.77, *p* < 0.001).

**Table 1:** Descriptive statistics about the young men (*N* = 76) included in our study.

	Mean	Min	Max	SD
Age (years)	20.5	18.8	24.4	1.1
Height (cm)	178.1	164.0	194.0	6.9
Weight (kg)	74.3	47.5	114.4	12.9
Body mass index (kg/m^2^)	23.4	17.4	34.7	3.5
Waist circumference (cm)	81.0	65.0	107.0	9.1
Relative fat mass (%)	14.6	0.1	34.4	7.7
Absolute fat mass (kg)	11.7	0.1	39.1	8.0
Skeletal muscle mass (kg)	30.7	21.3	39.2	3.7
Surface area (m^2^)	1.85	1.44	2.27	0.17
Volume (m^3^)	0.07	0.05	0.12	0.01
Point cloud (*n*)	491,104	425,691	594,259	33,138

Before discussing the results of our analysis, we exemplified in Fig. 1 the range of body shapes in our study sample with a roughness analysis at radius = 2 cm (R2) mapped onto the 3D surface models of a slim (BMI = 19.5; relative body fat = 2.5%), a muscular (BMI = 21.0; relative body fat = 4.3%), and an obese (BMI = 30.1; relative body fat = 34.4%) study participant and added the corresponding roughness frequency tables. All frequency table distributions were right skewed. A visual evaluation also revealed that in the most obese study subject, the absolute value of the Peak Class (mode) was higher than the peaks of the slimmest or the most muscular study subjects. Additionally, the location of the mode in the most obese participant was in a lower class (Class 10, using R2, radius = 2 cm) compared to the peaks of the slimmest participant (Class 16) or the most muscular participant (Class 12), i.e., most surface points of the obese participants were located within smoother surface areas.

In our first analysis step, we assessed the areal surface roughness frequency tables by linear regressions, using the basic measures of statistical dispersion SD, IQR, and mode for the estimation of %BF. Among all basic models, M1–M9 (details are given in Table 2), M5 (R2, IQR) showed the best explanatory variance (*R*^2^ = 0.55, *p* < 0.0001). All associations were negative (as expected). In our second analysis step, M12 (R5, IQR, mode) showed the best association with %BF among the stepwise regression models M10–M12 presented in Table 2. This model M12 showed a higher proportion of explained variation for %BF (*R*^2^ = 0.69, *p* < 0.0001) than the models with single parameters (M1–M9). The strongest contributions were coming from mode (AIC = 29.11), followed by IQR (AIC = 45.90). In our more explorative third analysis step (models M13–M15 in Table 2), arithmetic mean height (Sa) showed the overall best association with %BF. Model M14 (R2) showed the best association with %BF (*R*^*2*^ = 0.74, *p* < 0.001). All reported *R*^2^ are adjusted *R*^2^.

**Table 2:** Statistical data of all linear regression models (M1–M15) assessed in this study.

	Model	Radius (cm)	Explanatory variable(s)	*R*^2^ (adjusted)	*p* value	Regression equation
Analysis step 1						
(Basic models)	M1	1	SD	0.10	3.7850E−03	%BF = 53.947 − 4.73 × SD
	M2	2	SD	0.30	1.5700E−07	%BF = 85.9321 − 5.7820 × SD
	M3	5	SD	0.42	1.3600E−10	%BF = 93.9445 − 3.6815 × SD
	M4	1	IQR	0.22	1.2700E−05	%BF = 55.806 − 6.619 × IQR
	M5	2	IQR	0.55	7.6800E−15	%BF = 79.1618 − 5.6194 × IQR
	M6	5	IQR	0.54	1.7200E−14	%BF = 84.2318 − 2.6051 × IQR
	M7	1	Modus	0.50	5.1900E−13	%BF = 54.2206 − 6.7645 × Modus
	M8	2	Modus	0.46	9.9800E−12	%BF = 50.3813 − 3.1282 × Modus
	M9	5	Modus	0.43	6.8100E−11	%BF = 39.8219 − 0.9579 × Modus
Analysis step 2						
(Stepwise regressions)	M10	1	SD IQR Modus	0.58	2.5110E−14	%BF = 89.4293 − 5.7329 × Modus − 2.5295 × SD − 3.2478 × IQR
	M11	2	SD IQR Modus	0.68	2.2000E−16	%BF = 96.1587 − 1.6816 × Modus − 1.8972 × SD − 3.3880 × IQR
	M12	5	IQR Modus	0.69	1.3700E−11	%BF = 83.7314 − 0.6179 × Modus − 1.9780 × IQR
Analysis step 3						
(Explorative)	M13	1	Sa	0.66	2.2000E−16	%BF = 110.793 − 12.622 × Sa
	M14	2	Sa	0.74	2.2000E−16	%BF = 118.6964 − 7.4138 × Sa
	M15	5	Sa	0.73	2.2000E−16	%BF = 108.4183 − 2.9184 × Sa

In the models M1–M9, we assessed the areal surface roughness frequency tables by linear regressions, using the basic measures of statistical dispersion standard deviation (SD), interquartile range (IQR), and mode for the estimation of %BF. M5 (R2, IQR) showed the best explanatory variance (*R*^2^ = 0.55, *p* < 0.0001). M10–M12 are stepwise regression models, of which M12 (R5, IQR, mode) showed the best association with %BF (*R*^2^ = 0.69, *p* < 0.0001). In the models M13–M15 we assessed the association of the arithmetic mean height (Sa) and %BF, of which M14 (R2) showed the best association (*R*^2^ = 0.74, *p* < 0.001). All reported *R*^2^ are adjusted *R*^2^.

**Fig. 2:**
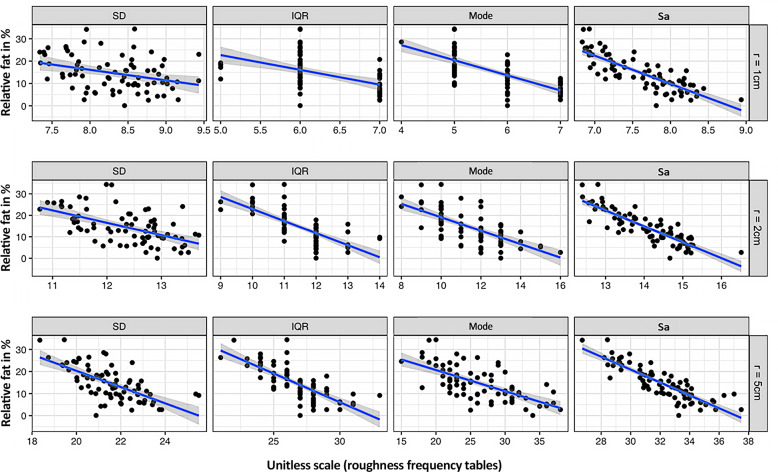
Scatter plots comparing BIA body fat measurements and statistical parameters of the frequency tables. Scatter plots with fitted linear regression lines, providing a visual comparison of BIA body fat measurements and areal surface roughness frequency tables computed using radii of 1, 2, and 5 cm based on standard deviation (SD), interquartile range (IQR) (Models M1–M6), location of the mode (Models M7–M9), and Sa (Models M13–M15).

Surfaces of study subjects with higher %BF showed more points lying in surface areas of lower roughness. SD and IQR were lower, consistent with a higher %BF (Fig. 2). Slimmer or more muscular study subjects’ body surfaces featured more accentuated details, represented by a broader distribution in the areal surface roughness frequency tables. In the linear and stepwise regression models, the explanatory variance was slightly lower compared to models using BMI, WC, or Sa (arithmetic mean height) (Table 2). The analysis of the areal surface roughness frequency tables, assessed by SD, IQR, and mode, as well as calculated Sa (arithmetic mean height), showed negative associations of all parameters with %BF. Overall, roughness distribution analyses, using radii greater than 1 cm, showed better associations with %BF. Best associations were achieved using a radius of 2 cm (R2) and second-best using 5 cm (R5).

## Discussion

Our feasibility study aimed at providing a proof of concept that areal body surface roughness characteristics may be used as a proxy for %BF. To the best of our knowledge, it is the first study analyzing the association between the areal roughness of the body’s surface and body composition. While the individual parameters of statistical dispersion (SD, IQR, and mode) in the calculated frequency tables only showed moderate associations with %BF, a stepwise regression analysis, considering multiple parameters, showed stronger associations. The best association (quantified by *R*^2^) with %BF was observed for Sa (*R*^2^ = 0.74), the arithmetic mean of roughness, which compared to other association studies of individual scanner-based measurements (e.g., circumferences, lengths, volumes) with aspects of body composition, and using comparable samples [17], represents a medium to strong association. However, somewhat stronger associations have been demonstrated when several scanner measurements were combined using different statistical methods [21].

The main limitation of the present study is that it is a technical proof of concept and not a detailed validation study requiring further external data. A detailed validation of the proposed method is indeed the next logical step for the future. Further, only young Swiss men aged 18–24 years were included in the study; however, the distribution of adipose tissue varies according to sex and age. Second, while the association with %BF was quite successful, the distinction between muscular and thin study subjects remained imprecise, as it is also the case for the BMI. Also, for our study, participants wore bathing caps and underpants during scanning, which created wrinkles and may have influenced the areal surface roughness distribution. We considered BIA a sufficiently accurate estimate of %BF for initial association tests in a feasibility study. However, additional and more differentiated data from more precise techniques (e.g., full-body MRI) may be required to fully understand the relationship between specific body surface parameters and underlying tissue composition.

In future investigations, we plan to focus on longitudinal intraindividual observations. In follow-up measurements, we shall, therefore, check whether changes in overall areal surface roughness coincide with changes in %BF. Depending on individual parameters, such as age, sex, or physical activity level, shifts in body compositions may follow distinct distribution patterns identifiable by areal surface roughness measurements.

## Conclusion

This study shows that 3D body surface roughness characteristics associate well with body composition and represent a viable new approach to improve today’s 3D scanner-based methods to assess body composition and obesity-associated health risks. Although the explanatory variance of some parameters evaluated in our approach was slightly lower than in comparable trials, this first evidence is an auspicious starting point for future studies. The arithmetic mean height reached medium to strong levels of explanatory variance for relative body fat compared to the BMI or WC. Further investigations may provide a more detailed understanding of the association between areal surface roughness characteristics and well-established approaches, such as BIA body fat estimation, by including larger and more diverse study cohorts or by focusing on particular body segments.

## Data Availability

The data that support the findings of this study are available from the corresponding author, PE, upon reasonable request.
